# HIV-1 Inhibits Phagocytosis and Inflammatory Cytokine Responses of Human Monocyte-Derived Macrophages to *P. falciparum* Infected Erythrocytes

**DOI:** 10.1371/journal.pone.0032102

**Published:** 2012-02-21

**Authors:** Louise E. Ludlow, Jingling Zhou, Emma Tippett, Wan-Jung Cheng, Wina Hasang, Stephen J. Rogerson, Anthony Jaworowski

**Affiliations:** 1 Department of Medicine (RMH), University of Melbourne, Post Office Royal Melbourne Hospital, Melbourne, Victoria, Australia; 2 Victorian Infectious Diseases Service, Royal Melbourne Hospital, Parkville, Victoria, Australia; 3 Centre for Virology, Burnet Institute, Melbourne, Victoria, Australia; 4 Department of Medicine, Monash University, Victoria, Australia; 5 Department of Immunology, Monash University, Victoria, Australia; London School of Hygiene and Tropical Medicine, United Kingdom

## Abstract

HIV-1 infection increases the risk and severity of malaria by poorly defined mechanisms. We investigated the effect of HIV-1_Ba-L_ infection of monocyte-derived macrophages (MDM) on phagocytosis of opsonised *P. falciparum* infected erythrocytes (IE) and subsequent proinflammatory cytokine secretion. Compared to mock-infected MDM, HIV-1 infection significantly inhibited phagocytosis of IE (median (IQR) (10 (0–28) versus (34 (27–108); IE internalised/100 MDM; p = 0.001) and decreased secretion of IL-6 (1,116 (352–3,387) versus 1,552 (889–6,331); pg/mL; p = 0.0078) and IL-1β (16 (7–21) versus 33 (27–65); pg/mL; p = 0.0078). Thus inadequate phagocytosis and cytokine production may contribute to impaired control of malaria in HIV-1 infected individuals.

## Introduction

Due to the high prevalence of HIV-1 in *Plasmodium falciparum* endemic regions such as sub-Saharan Africa, co-infections are common in pregnant women, affecting approximately 1 million pregnancies each year [1]. HIV-1 co-infection is associated with impaired control of malaria resulting in more frequent and higher peripheral and placental parasite densities [2]. HIV-1 infection increases the risk and severity of pregnancy-associated malaria by poorly defined mechanisms.

Primigravid women are at increased risk of placental malaria characterised by the accumulation of *P. falciparum* infected erythrocytes (IE) in the intervillous spaces of the placenta. Complications of malaria in pregnancy include severe anaemia and low infant birth weight. These complications are associated with monocyte accumulation in the maternal intervillous circulation of the placenta, termed intervillositis [3], and with increased placental blood TNF concentrations [4]. Monocytes and macrophages in the intervillous space frequently contain the malaria pigment haemozoin, and intact IE are also seen within these cells. This phagocytosis represents an important mechanism of controlling blood trophozoite-stage parasites and is enhanced by antibody opsonisation [5].

In the placenta, the principal target for opsonising antibody on the IE surface appears to be the variant surface antigen VAR2CSA, which mediates binding to chondroitin sulphate A (CSA) present on the placental syncytiotrophoblast [6]. Antibodies against VAR2CSA block placental sequestration and opsonise IE for phagocytic uptake. Antibodies to VAR2CSA develop with exposure during successive gravidities, and are associated with decreased prevalence and intensity of infection and with protection against low birth weight and severe maternal anaemia [7], [8], [9]. We have demonstrated that IgG opsonic activity in serum is associated with protection from treatment failure [10] and is gravidity dependent [11] in pregnant women in Malawi. The relative risk of malaria associated with HIV-1 infection is greatest in multigravidae [12], consistent with an effect on acquired antibody-dependent immunity. HIV-1 infection impairs development of opsonising antibodies to pregnancy-associated variant surface antigens including VAR2CSA [13] and we have demonstrated lower serum opsonic activity in multigravid women with malaria and HIV-1 co-infection [14]. Opsonising antibodies engage Fcγ receptors which promote phagocytic ingestion and induce kinase and transcription factor activation which orchestrates proinflammatory cytokine secretion [15], [16], [17]. Signalling mechanisms that result in this cytokine profile in response to intact IE are presently unknown, but clinical observations confirm that proinflammatory cytokines and β chemokines are secreted by intervillous macrophages and monocytes in response to IE [18]. This alteration in cytokine balance is important for clearance of IE from the placenta, but it is also associated with maternal anaemia and premature delivery [4], [19], [20], [21], [22].

We hypothesised that HIV-1 may inhibit opsonic phagocytosis thereby impairing IE clearance resulting in increased susceptibility of multigravid women to pregnancy-associated malaria. To further our understanding of the mechanisms by which HIV-1 co-infection impairs immunity to malaria in pregnancy, we investigated the effects of *in vitro* HIV-1 infection on phagocytic uptake and cytokine secretion by monocyte-derived macrophage (MDM) in response to opsonised CS2-IE (an established model for CSA binding placental strains of *P. falciparum*). Compared to mock-infected MDM, HIV-1 infection significantly inhibited phagocytosis of opsonised IE without altering MDM viability, and decreased secretion of IL-6 and IL-1β. These results may explain the increased risk and severity of maternal malaria in the setting of HIV-1 infection.

## Materials and Methods

### Isolation of monocytes and culture of MDM

Human PBMC were obtained from Buffy Coats separated from volunteer blood donations (Australian Red Cross Blood Service, Southbank, Victoria, Australia) using Ficoll-Paque™ density gradient centrifugation. Monocytes were isolated from this cell fraction by counter-current elutriation using a Beckman J-6M/E centrifuge equipped with a JE 5.0 rotor. Preparations yielded 50–100×10^6^ monocytes and were 90–92% pure with a viability of >98% determined by flow cytometry and trypan blue exclusion techniques respectively (data not shown). Monocyte-derived macrophages (MDM) were prepared by culturing freshly isolated monocytes adhered to plastic in Iscove's modified Dulbecco's medium (Invitrogen) containing 10% non-malaria immune heat-inactivated human serum obtained from the Red Cross Blood Service, Sydney, Australia supplemented with 2 mM glutamine, 100 U/mL penicillin G and 100 µg/ml streptomycin sulphate (IH10 medium). MDM were cultured for 14 days at a cell density of 50,000 per well in 96-well plates and 2.5×10^5^ per well in 24-well plates.

### Infection of MDM with HIV-1_Ba-L_

HIV-1_Ba-L_ was routinely passaged in human MDM and purified from culture media by centrifugation (60 min, 4°C, 150,000×g) through a 20% sucrose cushion in Ca^2+^/Mg^2+^ PBS. Viral stocks were analysed by micro reverse transcriptase (RT) assay which quantifies the enzymatic activity of HIV-1 RT by measuring the incorporation of [^33^P]-labelled dTTP into DNA from a synthetic RNA primer/template, and is expressed as counts per minute of radioactivity incorporated into DNA per hour per microlitre of culture medium [23]. Laboratory stocks of HIV-1_Ba-L_ were verified by comparing the sequence of the hypervariable V3 loop of the Env gene to that of the reference HXB2 strain, amplified using the following primers: Forward: 5′- ACAATGYACACATGGAATTARGCCA-3′, Reverse: 5′-AGAAAAATTCYCCTCYACAATTAAA-3′. MDM were infected with HIV-1_Ba-L_ at a multiplicity of infection (MOI) of between 0.1 and 1.0 and cultured for a further 7 days before use in phagocytosis or cytokine assays. Where indicated, MDM were primed with IFNγ (100 ng/mL, R&D Systems) 5 days after HIV-1_Ba-L_ infection to allow 48 hr priming of macrophages before exposure to phagocytic targets. Culture medium was changed every 3–4 days and infection was monitored by measuring RT activity in culture medium 7 days post HIV-1_Ba-L_ infection; using this protocol, the percentage of MDM productively infected with HIV-1_Ba-L_ was 30–70% [24], [25]. MDM viability was measured using reduction of sodium 3′-[1-(phenylaminocarbonyl)-3,4-tetrazolium]-bis(4-methoxy-6-nitro) benzene sulphonic acid (XTT) as an indicator according to the manufacturer (Roche, Cell Proliferation Kit II).

### Culture and purification of parasitised erythrocytes

The laboratory-adapted *P. falciparum* line CS2 resembles placental-type isolates based upon VAR2CSA expression, binding to CSA and recognition by serum in a pregnancy and gravidity-specific manner. CS2 was cultured in unexpired human group O^+^ erythrocytes (Australian Red Cross Blood Service). Cells were maintained at 5–12% parasitemia in RPMI 1640-HEPES medium supplemented with 0.25% AlbumaxII (Gibco) and 0.2% w/vol NaHCO_3_. Cultures were synchronized by gelatine flotation every 1 to 2 weeks and adhesion to CSA was regularly checked to ensure high level binding. Cultures were tested regularly to exclude Mycoplasma contamination. Trophozoite-stage parasites were purified by density gradient centrifugation using layers of 80%, 60% and 40% Percoll in supplemented RPMI 1640-HEPES. Purified IE collected from the 60% layer were washed three times and resuspended in supplemented RPMI. Preparations were analysed microscopically for stage and contamination by uninfected erythrocytes, and a purity between 92–95% was routinely obtained.

### Opsonisation of CS2 Trophozoites

IE were left unopsonised or opsonised with 9% heat-inactivated pooled patient serum (PPS) from Malawian HIV-uninfected pregnant women with malaria, for 30 min at room temperature as described [14]. IE were examined microscopically to verify that opsonisation at these concentrations did not induce agglutination. Opsonised IE were washed and resuspended in PBS at 1×10^8^ per mL and used immediately.

### Measurement of phagocytosis and cytokine secretion

IE were added at 1×10^6^ per well to MDM cultured in 96-well plates (a target to cell ratio of 20∶1) and incubated for 1 hr. Phagocytosis was determined by measuring internalised haemoglobin using a colourimetric assay as described [14]. The haemoglobin content was converted to equivalents of IE ingested by reference to a standard curve of known amounts of IE from the same preparation, and phagocytosis was expressed as a phagocytic index representing IE ingested per 100 MDM. At the indicated time point, media from triplicate wells was collected and pooled, then analysed for cytokine secretion using cytokine bead array (BD Biosciences, Human Inflammatory Cytokine Kit).

### Measurement of cytokine gene expression by quantitative real time PCR

MDM were cultured in 24-well plates and exposed to IE for various times then lysed in lysis buffer A (0.1 M Tris HCl, pH 7.5 containing 1% lithium dodecyl sulphate, 0.5 M LiCl, 10 mM EDTA, 5 mM DTT) to extract total cellular RNA. Cellular mRNA was isolated from extracts using oligo(dT) magnetic beads (GenoPrep™, GenoVision), then cDNA was prepared using a Transcriptor First Strand cDNA Synthesis Kit (Roche) followed by amplification of cytokine cDNAs by quantitative real time PCR in Brilliant® SYBR® Green qPCR Master Mix (Stratagene) using the following primer sets [26]: TNF: Forward: 5′ CCCCAGGGACCTCTCTCTAATC 3′, Reverse: 5′ GGTTTGCTACAACATGGGCTACA 3′, NM_000594; IL-1β: Forward: 5′ CCTGTCCTGCGTGTTGAAAGA 3′, Reverse: 5′ GGGAACTGGGCAGACTCAAA 3′, NM_000576; IL-6: Forward: 5′ CAATCTGGATTCAATGAGGAGAC 3′, Reverse: 5′ CTCTGGCTTGTTCCTCACTACTC 3′, NM_000600.

Messenger RNA levels were determined by quantitative real time PCR (Stratagene MX-3000) using the comparative threshold method with GAPDH as a reference gene. PCR conditions were 95°C for 10 min and 40 cycles of 95°C for 30 sec, 58°C for 30 sec and 72°C for 30 sec. In all cases, amplification products from the last cycle were resolved by 1% agarose gel electrophoresis containing 1× SYBR Safe® DNA gel stain (Invitrogen) and visualised using a Gel Doc XR Molecular Imager (Bio-Rad) to verify single amplification products.

### Statistical Analysis

Statistical comparisons between groups were performed using Mann-Whitney non-parametric U test or for paired comparisons a Wilcoxon signed rank test. All statistical analysis was carried out using Prism 5.0 software (GraphPad Software). Significance was defined as a probability value below 0.05

### Ethics

Sera used to produce the positive pool were collected as part of studies approved by the College of Medicine Research Ethics Committee, Blantyre, Malawi and the Melbourne Health Human Research Ethics Committee, Melbourne. Informed written consent was obtained from all participants involved in this study.

## Results

### HIV-1 inhibits antibody dependent uptake of CS2 infected erythrocytes

**Figure 1:**
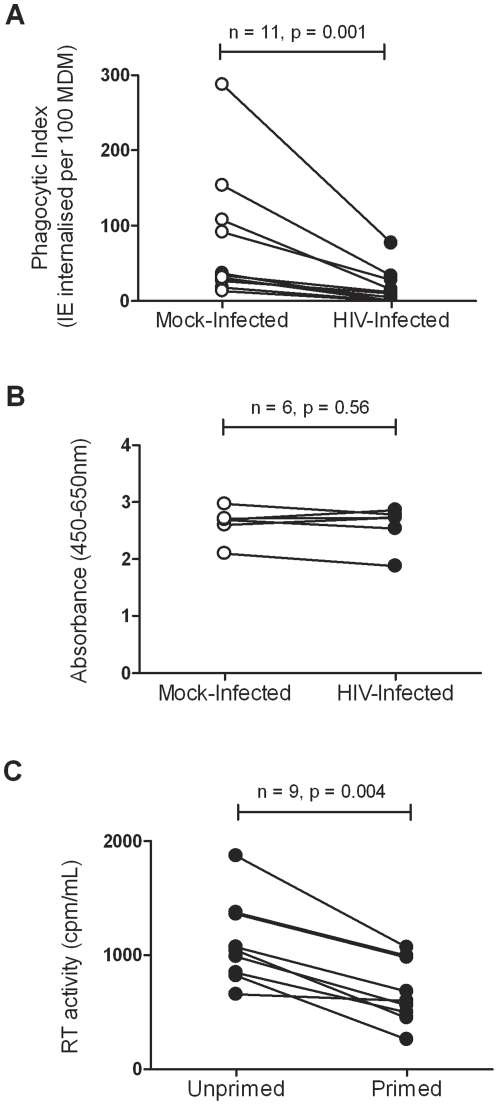
HIV-1 infection of MDM inhibits phagocytosis of antibody opsonised CS2 infected erythrocytes. (**A**). MDM from 11 donors were cultured for 5 days and infected with HIV-1_Ba-L_ (•) at a MOI of between 0.1 and 1.0 or mock-infected (○) then cultured for an additional 7 days. IE opsonised with 9% PPS were added at a 20∶1 ratio and phagocytosis was measured at 1 hour. Phagocytic index represents the number of ingested IE per 100 MDM. Statistical comparisons were performed in a pair wise manner using the Wilcoxon matched pairs test. (**B**). In 6 selected cultures analysed for phagocytosis, XTT assay was used to measure viability of MDM infected with HIV-1_Ba-L_ (•) or mock-infected (○) MDM (3 mg/mL XTT, 4 hr, absorbance was read at 450 nm using a reference wavelength of 650 nm). (**C**). Effect of IFNγ on HIV-1_Ba-L_ production by MDM. MDM were cultured in triplicate in 96-well plates for 5 days, infected with HIV-1_Ba-L_ for a further 5 days then primed with 100 ng/mL IFNγ where indicated. After 72 hrs media from triplicate wells was collected and analysed by micro RT assay. Comparisons between unprimed and primed MDM were made using the Wilcoxon matched pairs test and a significant difference (p<.05) is indicated.

The effect of HIV-1 infection on macrophage ingestion of opsonised IE was investigated. Phagocytosis assays were performed using CS2-IE opsonised with PPS as targets. MDM generated from eleven different donor monocyte preparations were infected or mock-infected with the laboratory-adapted macrophage-tropic strain HIV-1_Bal-L_. HIV-1 infection significantly inhibited (p = 0.001) ingestion of opsonised IE (9.6 IE internalised per 100 HIV-1_Ba-L_-infected MDM (IQR: 0–28) compared to 34 IE internalised per 100 mock-infected MDM (IQR: 27–108); Figure 1A). HIV-1_Ba-L_ infected or mock-infected MDM did not phagocytose uninfected erythrocytes or unopsonised IE (data not shown) and similar results were obtained using IFNγ primed MDM (data not shown). In selected experiments, MDM viability was assessed to verify that inhibition of phagocytosis was not due to effects of HIV-1_Bal-L_ infection on cell viability. In six independent experiments using MDM prepared from different donor monocytes, no significant difference (p = 0.56) in cell viability was observed between mock-infected and HIV-1_Bal-L_ infected MDM (Figure 1B). As expected, due to its antiviral effects, IFNγ treatment reduced HIV-1_Bal-L_ production measured at day seven post-infection from a median of 1040 cpm in the absence of priming to 599 cpm with priming (p = 0.004; Figure 1C).

### HIV-1 alters proinflammatory cytokine secretion by MDM in response to opsonised IE

**Figure 2:**
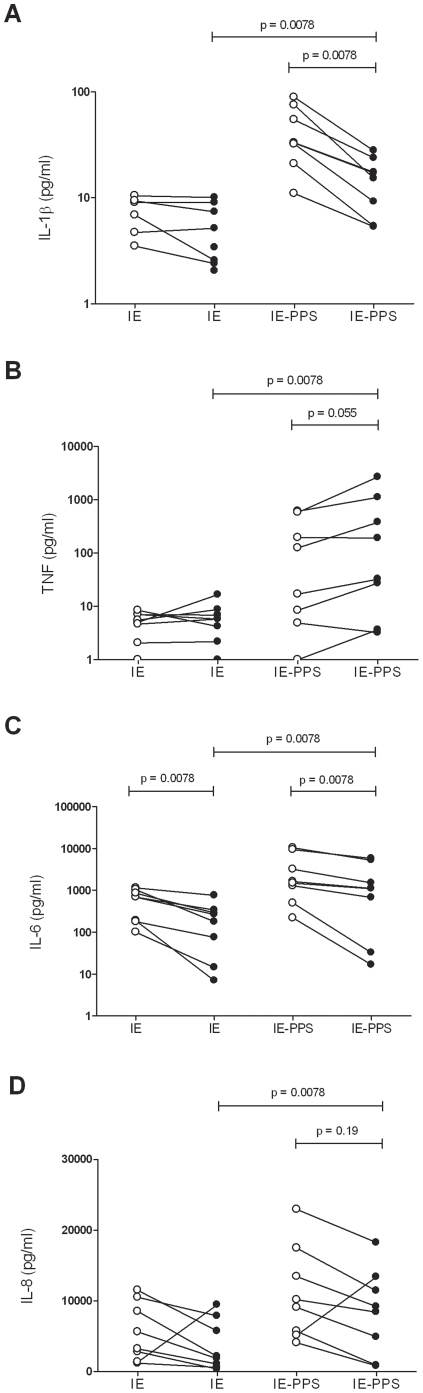
The effect of HIV-1 on cytokine secretion of MDM exposed to antibody opsonised IE. MDM from 8 donors cultured as described in Figure 1 were infected with HIV-1_Ba-L_ (•) or mock-infected (○) and primed for 48 hrs with IFNγ then exposed to unopsonised (IE) or IE opsonised with 9% pooled immune serum (IE-PPS) at a target to cell ratio of 20∶1 for 24 hrs followed by collection of culture medium for (**A**) IL-1β, (**B**) TNF, (**C**) IL-6 and (**D**) IL-8 cytokine secretion analysis. Comparisons of cytokine secretion between cultures infected with HIV-1_Ba-L_ exposed to IE or IE-PPS, and between HIV-1_Ba-L_ infected and uninfected cultures exposed to IE-PPS were made using the Wilcoxon matched pairs test.

The effect of HIV-1 infection on cytokine secretion following opsonised CS2-IE ingestion by MDM was then assessed. Uptake of opsonised IE significantly increased secretion of TNF, IL-6, IL-1β and IL-8 in both HIV-1_Ba-L_ infected and mock-infected MDM primed with IFNγ (Figure 2) or unprimed (data not shown). In response to opsonised IE, HIV-1 infection significantly reduced IL-6 release (1,116 pg/mL (IQR: 352–3,387) compared to 1,552 pg/mL (IQR: 889–6,331); p = 0.0078) and IL-1β release (16 pg/mL (IQR: 7–21) compared to 33 pg/mL (IQR: 27–65); p = 0.0078) from mock-infected IFNγ primed MDM (Figure 2A and 2C). Similar results were observed using unprimed MDM where in response to opsonised IE, HIV-1 infection significantly reduced IL-6 release (410 pg/mL (IQR: 40–1985) pg/mL compared to 798 pg/mL (IQR: 276–2671); p = 0.015) and IL-1β release (14 pg/mL (IQR: 5–24) compared to 31 pg/mL (IQR: 8–73); p = 0.039) from mock-infected unprimed MDM (data no shown).

HIV-1 infection had variable effects on TNF secretion in response to IE-PPS phagocytic targets (112 pg/mL (IQR: 16–745) compared to 71 pg/mL (IQR: 7–388); p = 0.055) using IFNγ primed MDM and (8 pg/mL (IQR: 3–42) compared to 5 pg/mL (IQR: 3–9); p = 0.078) in unprimed MDM (Figure 2B and data not shown). No significant decrease in IL-8 secretion was observed in both primed (p = 0.19) and unprimed MDM (p = 0.46; Figure 2D and data not shown). Secretion of IL-12p70 or IL-10 was not detected in response to IE-PPS (data not shown).

**Figure 3:**
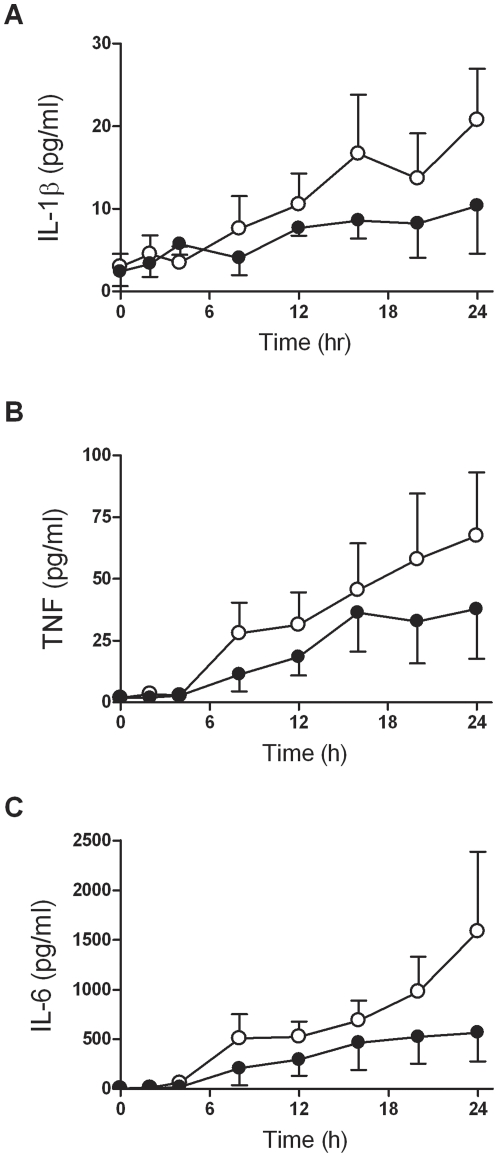
Time course of the effect of HIV-1 on cytokine secretion of MDM exposed to antibody opsonised IE. MDM from 3 donors cultured as described in Figure 1 were infected with HIV-1_Ba-L_ (•) or mock-infected (○) and primed for 48 hrs with IFNγ then exposed to IE opsonised with 9% pooled immune serum (IE-PPS) at a target to cell ratio of 20∶1. At the indicated time points culture supernatants were collected for (**A**) IL-1β, (**B**) TNF and (**C**) IL-6 cytokine measurement. Data are means of MDM derived from 3 donors plus-minus SEM. Statistical analysis was carried out using the generalised estimating equations method which indicated that mock-infected MDM secreted significantly higher levels of cytokines compared to HIV-1 infected MDM (p = 0.026, IL-1β; p = 0.047, TNF; p = 0.019, IL-6).

The effect of HIV-1 infection on the kinetics of cytokine secretion following opsonised CS2-IE ingestion by MDM prepared from 3 independent donors was assessed (Figure 3). No difference in IL-1β, TNF and IL-6 secretion was observed in response to IE-PPS phagocytic targets at 2 hours and 4 hours in HIV-1 infected MDM compared to mock-infected MDM. From 8 hours onwards a time-dependent decrease in IL-1β, TNF and IL-6 accumulation in culture medium was detected in HIV-1 infected MDM exposed to IE-PPS (Figure 3).

### HIV-1 alters proinflammatory cytokine mRNA production

**Figure 4:**
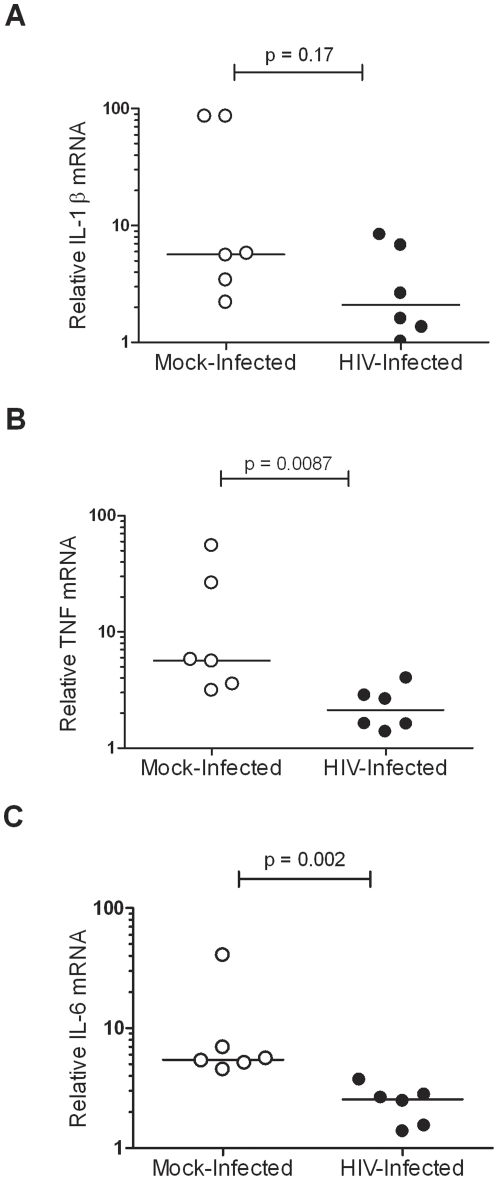
The effect of HIV-1 on cytokine mRNA levels of MDM exposed to antibody opsonised IE. MDM from 6 donors cultured in 24-well plates were infected with HIV-1_Ba-L_ (•) or mock-infected (○) and primed for 48 hrs with IFNγ then exposed to IE opsonised with 9% pooled immune serum (IE-PPS) at a target to cell ratio of 20∶1 for 2 hrs followed by preparation of RNA and cDNA for analysis of (**A**) IL-1β, (**B**) TNF and (**C**) IL-6 cytokine mRNA levels by qPCR. Comparisons of mRNA levels between cultures infected with HIV-1_Ba-L_ or mock-infected exposed to IE-PPS were made using the Mann-Whitney non-parametric U test.

To determine whether reduced cytokine secretion was due to HIV-1 mediated decrease of mRNA expression, HIV-1 infected and uninfected MDM from six additional donors were exposed to IE-PPS for 1 hour, 2 hours or 4 hours and the levels of IL-1β, TNF and IL-6 transcripts were measured by qPCR. No significant decrease in IL-1β mRNA was observed at 2 hours (p = 0.17; Figure 4A), 1 hour (p = 0.31; data not shown) or 4 hours (p = 0.48; data not shown) in HIV-1_Ba-L_ infected MDM compared to mock-infected MDM exposed to IE-PPS. HIV-1 infection had variable effects on TNF mRNA levels in response to IE-PPS phagocytic targets. A significant decrease in TNF mRNA was observed at 2 hours (p = 0.0087; Figure 4B) but not at 1 hour (p = 0.13; data not shown) or 4 hours (p = 0.39; data not shown). In response to opsonised IE, HIV-1 infection significantly reduced IL-6 mRNA at 2 hours (p = 0.002; Figure 4C), 1 hour (p = 0.026; data not shown) and 4 hours (p = 0.04; data not shown).

## Discussion

In this study we reveal that HIV-1 infection of MDM inhibits phagocytosis and decreases proinflammatory cytokine secretion in response to IE. Insufficient phagocytosis and consequent cytokine production in the context of HIV-1 infection may contribute to the increased risk and severity of pregnancy-associated malaria.

This study demonstrates that HIV-1 infection *in vitro* significantly impairs ingestion of antibody opsonised IE. In agreement, previous research has reported reduced FcγR-dependent phagocytosis of IgG-coated erythrocytes by HIV-infected MDM [23], [27]. We have previously demonstrated a potential mechanism for defective FcγR-dependent phagocytosis. The macrophage Fcγ receptor family (FcγRI, FcγRII and FcγRIIIa) are responsible for recognition of IgG-opsonised targets, activating signal transduction pathways via tyrosine kinases of the Syk/ZAP70 family leading to target ingestion and cytokine secretion. HIV-1 infection results in reduced intracellular protein levels of FcRγ, the signalling adaptor protein and chaperone required for FcγRI and III expression and function [23]. Inhibition of subsequent downstream phosphorylation of Hck and Syk tyrosine kinases is evident in HIV-infected MDM undergoing Fcγ receptor-mediated phagocytosis. Thus impaired FcγR-mediated signalling in HIV-1 infected MDM may explain the decreased phagocytosis of opsonised IE observed in our analysis. However, decreased IL-6 secretion was also observed in response to unopsonised IE which are not ingested by MDM. This suggests that HIV impairs other pathways required for proinflammatory cytokine secretion in addition to those activated by Fcγ receptors.

Inappropriate regulation of inflammatory cytokines by HIV-1 infected macrophages in the context of high IFNγ concentrations may prevent an adequate immune response to parasitemia. Studies examining cytokine levels in a cohort of HIV-1 infected women with placental malaria are justified. In partial agreement with our observations, IFNγ, IL-4 and IL-10 production by intervillous blood mononuclear cells was impaired in HIV infected women with placental malaria [28]. Our laboratory has identified a mechanism that may explain these ex vivo observations. HIV-1 infection impairs development of opsonising antibodies to pregnancy-associated variant surface antigens including VAR2CSA [11] and lowers the capacity of multigravid pregnant women to opsonise IE [7], [12]. This is expected to decrease phagocytic parasite clearance in these women. The present study shows that HIV potentially affects the ability of the phagocytes to ingest opsonised IE, which would be expected to act in synergy with decreased plasma opsonic activity to impair clearance of blood stage parasites.

The reduction in IL-6 cytokine production derived from HIV-1 infected MDM exposed to IE-PPS appears to be at the level of mRNA expression. We investigated activation of NF-κB signalling in HIV-1 infected MDM stimulated with opsonised IE but did not observe any change in this pathway compared to mock-infected MDM (LEL and JZ, data not shown). The question remains as to why cytokine mRNA expression is decreased by HIV-1 infection. Study of the signalling mechanism responsible for the observed decrease in cytokine secretion is warranted.

We have demonstrated that HIV-1 inhibits ingestion of IE and subsequent proinflammatory cytokine production by macrophages which may explain the increased risk and severity of maternal malaria in the setting of HIV-1 infection. HIV infection therefore impairs immunity to placental malaria at multiple levels. Given that HIV co-infection is a major determinant of susceptibility to maternal malaria, control of HIV with antiretroviral drugs is critical to minimising the burden of maternal malaria. Whether effective control of HIV viremia by antiretroviral therapy restores immunity to maternal malaria is presently unknown.
